# Profiling of Altered Metabolomic States in *Bidens pilosa* Leaves in Response to Treatment by Methyl Jasmonate and Methyl Salicylate

**DOI:** 10.3390/plants9101275

**Published:** 2020-09-27

**Authors:** Anza-Tshilidzi Ramabulana, Paul A. Steenkamp, Ntakadzeni E. Madala, Ian A. Dubery

**Affiliations:** 1 Department of Biochemistry, Research Centre for Plant Metabolomics, University of Johannesburg, Auckland Park, Johannesburg 2006, South Africa; 201404841@student.uj.ac.za (A.-T.R.); psteenkamp@uj.ac.za (P.A.S.); ntaka.madala@univen.ac.za (N.E.M.); 2 Department of Biochemistry, University of Venda, Thohoyandou 0950, South Africa

**Keywords:** *Bidens pilosa*, caftaric acid, chicoric acid, caffeoylquinic acid, chlorogenic acid, flavonoids, metabolomic profiling, methyl jasmonate, methyl salicylate, phytohormones

## Abstract

*Bidens pilosa* (*Asteraceae*) is an edible medicinal plant with many bioactivities reported to have a health-beneficial role in controling various diseases. Though *B. pilosa* contain a diverse array of natural products, these are produced in relatively low concentrations. A possible way to enhance secondary metabolite production can be through the use of elicitors. Here, the effects of exogenous treatments with two signal molecules—methyl jasmonate (MeJA) and methyl salicylate (MeSA)—on the metabolomic profiles of *B. pilosa* leaves were investigated. Plants were treated with 0.5 mM of MeJA or MeSA and harvested at 12 h and 24 h. Metabolites were extracted with methanol and separated on an ultra-high performance liquid chromatography system hyphenated to quadrupole time-of-flight mass spectrometry detection. Data was subjected to multivariate statistical analysis and modeling for annotation of metabolites. Hydroxycinnamic acid (HCA) derivatives, such as caffeoylquinic acids (CQAs), tartaric acid esters (chicoric acid and caftaric acid), chalcones, and flavonoids were identified as differentially regulated. The altered metabolomes in response to MeSA and MeJA overlapped to a certain extent, suggestive of a cross-talk between signaling and metabolic pathway activation. Moreover, the perturbation of isomeric molecules, especially the *cis* geometrical isomers of HCA derivatives by both treatments, further point to the biological significance of these molecules during physiological responses to stress. The results highlight the possibility of using phytohormones to enhance the accumulation of bioactive secondary metabolites in this plant.

## 1. Introduction

*Bidens pilosa* L. (*B. pilosa*) is a cosmopolitan weed occurring mostly in hot tropical areas including sub-Saharan Africa, where it is a widely consumed leafy green vegetable source [1,2]. This plant is not only recognized for its nutritional value, but also for its pharmacological and bio-medical importance [3,4]. A diverse group of important compounds have been identified in *B. pilosa* such as aliphatics, terpenoids, tannins, alkaloids porphyrins, and phenylpropanoids including hydroxycinnamic acids (HCAs) and derivatives, chalcone okanins and flavonoids [3,5,6,7]. Some phenylpropanoids that have been reported in *B. pilosa* include a wide range of chlorogenic acids (CGAs) [8]. The health benefits of CGAs and flavonoids have been recently reviewed [9,10].

These secondary metabolites are the biosynthetic products of metabolic pathways that are responsive to environmental stressors or signal molecules associated with adaptation to changing environmental conditions that can affect the phytochemical composition [11]. In response to stress, levels of secondary metabolite phenolics are generally increased [12]. These phenolics are biosynthesized by the pentose phosphate-, shikimate- and the phenylpropanoid pathways and comprise of a common C6-C3 carbon skeleton with a hydroxylated benzene ring. Subclasses include simple phenols, which consist of coumarins and phenolic acids (HCAs and hydroxybenzoic acids) and polyphenols, which consist of flavonoids and tannins [13,14,15,16]. Several of these secondary stress-related metabolites act as antioxidants [17] and are associated with anti-microbial defenses or act as chemical deterrents to inhibit attack by insects and grazing animals [13,18]. For example, CGAs serve as precursors for wound-induced polyphenolic barriers such as ligin and suberin [19]. Isoflavonols and flavonoids are also induced as anti-microbials and protectants against UV radiation [12,20,21].

Methyl jasmonate (MeJA) and methyl salicylate (MeSA) are signal molecules of plant innate immunity and plant defense networks that have an effect on the physiological and biochemical processes that may trigger or enhance the production of various secondary metabolites [22]. MeSA is a derivative of salicylic acid (SA) produced through the action of methyltransferases on SA (a key compound derived from the shikimic acid pathway) and is a significant constituent in defense signaling cascades related to systemic acquired resistance (SAR) [23]. Similarly, MeJA is a volatile phytohormone (derived from jasmonic acid (JA) via the octadecanoid pathway), which is also a key mediator in the induced systemic resistance (ISR) response of plants [24]. The JA and SA defense signaling cross-communicate to fine tune defense responses. The former is induced to mediate defense against herbivores and necrotrophic pathogens whilst the SA pathway is induced in response to biotrophic pathogens [25,26]. These pathways are usually mutually antagonistic as each induce a different set of response genes to a particular pathogen [27]. The reciprocal antagonism of these pathways has been extensively described in *Arabidopsis thaliana*, where SA results in downregulation of JA-responsive genes and similarly a SA-mediated suppression of genes encoding key enzymes in JA biosynthesis has been described [26]. However, JA and SA pathways have also been described to act synergistically in *Nicotiana tabacum* when both hormones were applied at low (10–100 µM) concentrations, and enhancement of gene expression of genes associated JA and SA signaling was observed [28].

Exogenous treatment of plants with phytohormones is conceptually similar to triggering the reponses to abiotic or biotic stressors, as they induce defense-related metabolic pathways, thereby altering the metabolic profiles related to secondary metabolism [29]. Although MeJA and MeSA have been shown to induce the production of secondary metabolites such as phenolics in other plants [30], the effects of these signaling compounds have not been investigated in *B. pilosa.* Here, we report on a metabolomic investigation performed on methanolic extracts from leaf tissues of *B. pilosa* that were treated with MeJA and MeSA and harvested at time intervals of 12 h and 24 h. The results indicate alterations of the metabolome of *B. pilosa* in response to treatment with these phytohomones. Additionally, the profiling of metabolites in *B. pilosa* leaf tissues under exogenous treatment provides an understanding of the interaction between SA- and JA-regulated networks on the altered metabolomes as effected by MeJA and MeSA.

## 2. Results and Discussion

### 2.1. Profiling of Secondary Metabolites in Treated Leaves of Bidens Pilosa

MeJA and MeSA are signaling molecules that are produced in plants for the development and modulation of resistance to insects and pathogens [31]. Exogenous applications of MeJA and MeSA to plants imitate responses similar to that when plants are attacked by pathogens or herbivores and can enhance the production of secondary plant metabolites [30,32,33]. A common modification of plant secondary metabolites is methylation, catalyzed by various methyltransferases. Physiological and genetic analyses showed that reversible methylation of phytohormones participates in regulating various biological processes in plants, including stress responses and defense reactions. Phytohormones such as SA and JA are methylated by a subset of the SABATH family of methyltransferases, members of which have been reported to be responsive to to herbivores and pathogen infection [34].

**Figure 1:**
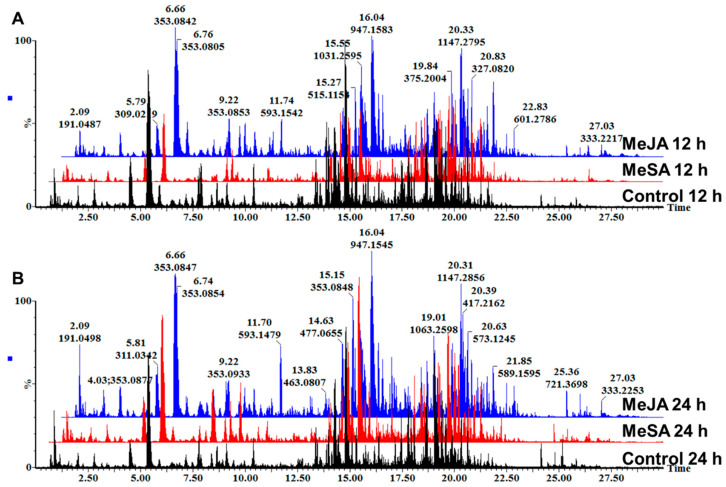
Ultra-high performance liquid chromatography, quadrupole time-of-flight mass spectrometry (UHPLC-qTOF-MS) base peak intensity (BPI) chromatograms in ESI-negative ionization mode of methanol extracts of *Bidens pilosa* leaf tissues treated with 0.5 mM methyl salicylate (MeSA) and 0.5 mM methyl jasmonate (MeJA) and harvested at 12 h (**A**) and 24 h (**B**) time intervals compared to the controls harvested at the respective times.

Here, MeJA and MeSA were infiltrated as elicitors into *B. pilosa* leaf tissues, with the aim to investigate the effects of these defense-related molecules on the recently described metabolome of *B. pilosa* [8]. The base peak intensity (BPI) chromatograms (Figure 1) of extracts from leaf tissues treated with MeJA and MeSA and harvested at 12 h (Figure 1A) and 24 h (Figure 1B) time intervals show minor differences as can be seen from the number of peaks and relative peak intensities. This required the employment of unsupervised explorative multivariate statistical analysis to visualize systematic trends within the data sets and the use of supervised multivariate statistical analysis to identify discriminant features differentiating between the treatments with the signaling molecules and the controls.

### 2.2. Multivariate Statistical Analysis of Extracts from Treated Plant Leaves

#### 2.2.1. Unsupervised Multivariate Statistical Analysis

**Figure 2:**
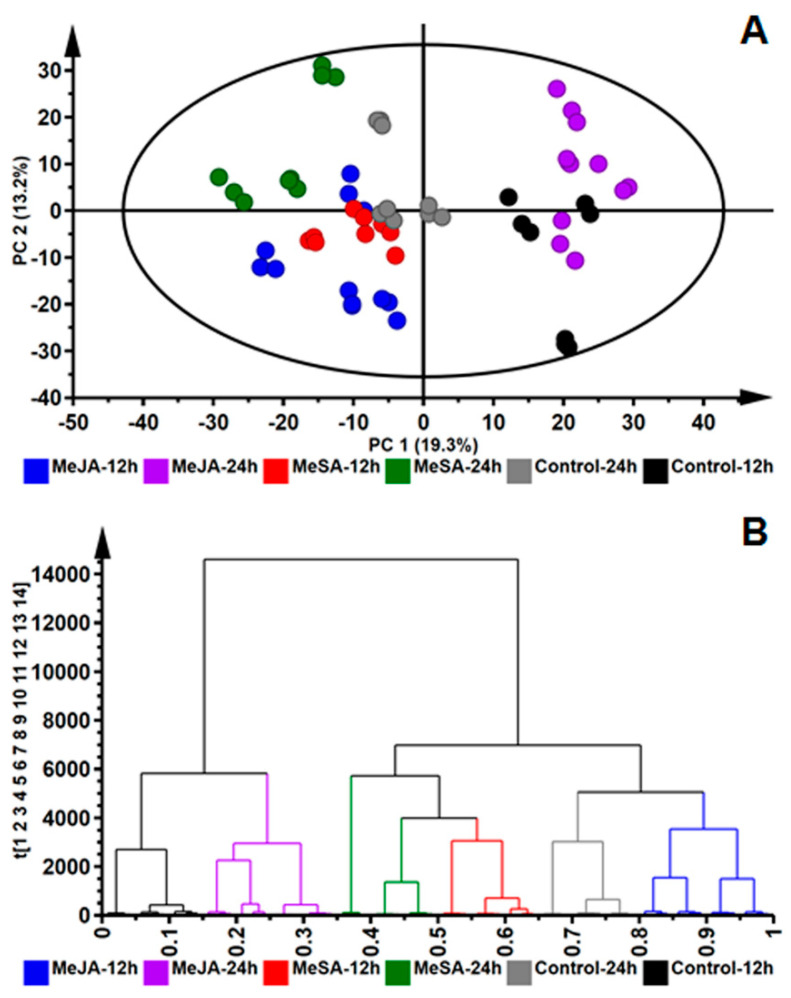
Unsupervised exploratory statistical analysis of *Bidens pilosa* leaf tissues infiltrated with 0.5 mM MeJA and MeSA and the non-treated control samples at 12 h and 24 h time points: (**A**) a principal components (PC) scores scatter plot of the *Pareto*-scaled data set. PC 1 and PC 2 of the 14-component model explained 32.5% of the variation. The quality parameters of the model were: explained variation/goodness of fit R^2^ = 0.781 and the predictive variance Q^2^ = 0.546. The ellipse indicates Hotelling’s T^2^ at 95% confidence interval. (**B**) The hierarchical cluster (HC) analysis plot shows the hierarchical structure of the data in a dendrogram format, showing treatment-specific clustering.

To explore and visualize the data sets generated by the UHPLC-MS analyses of methanol leaf extracts of plants treated with MeJA and MeSA, unsupervised multivariate statistical analysis was performed. A PC analysis model (Figure 2A) was constructed which reduced the dimensionality of the data and explained the variance within the data sets as mutually orthogonal PCs [35]. The computed model was based on 14 components with 32.5% of the variation accounted for by PC 1 and PC 2. The model was statistically adequate given the explained variation R^2^ = 0.781 and the predictive variance Q^2^ = 0.546. Regardless of the relatively minor differences observed in the BPI chromatograms, the PC scores plot indicated treatment-specific and time-dependent clustering [36].

Furthermore, a HC plot (Figure 2B) indicated intrinsic similarities and differences within the datasets. As observed in the HC dendrogram (Figure 2B), two major nodes are observed, indicating differences between the treated leaf extracts and the respective control samples. The first major node (Figure 2B, left-side) indicate that leaves treated with 0.5 mM MeJA and harvested at the 24 h time point (MeJA_24 h, purple lines) are more closely related to control samples harvested at the 12 h time point (Control_12 h, black lines). This may be a reflection that leaves treated with MeJA begin to return to metabolic homeostasis, 24 h post-treatment. On the second major node, extracts from leaves treated with 0.5 mM MeSA and harvested at 12 h and 24 h (MeSa_12 h, red lines and MeSA_24 h, green lines) clustered close to each other, suggesting similar metabolomes. These were also observed to form part of the cluster containing extracts from leaves treated with MeJA and harvested at 12 h (blue lines).

Together, the PC and HC analyses could indicate that the MeSA and MeJA treatments applied to *B. pilosa* leaves may have induced similar metabolic pathways, albeit with possible different kinetics. In order to annotate and identify the metabolites responsible for the differences observed and to derive comparative biological insights into the metabolic activation effected by the two phytohormones, further investigations involving supervised multivariate statistical analysis were conducted.

#### 2.2.2. Supervised Multivariate Analysis and Annotation of Metabolites

**Figure 3:**
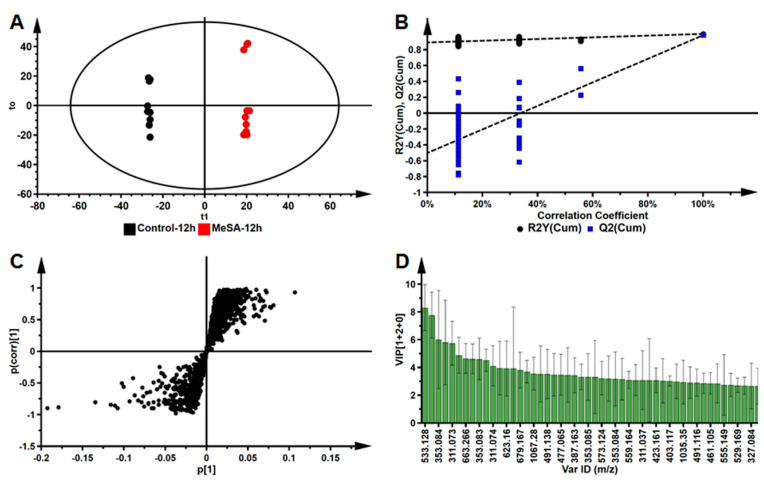
An orthogonal projection to latent structures discriminant analysis (OPLS-DA) model of control (black) and 0.5 mM MeSA (red) treated leaf extracts at 12 h post-treatment. (**A**) A scores plot summarizing the relationship between the two conditions. (**B**) A permutation test plot (n = 50) in response to the OPLS-DA plot indicated in (**A**), with quality parameters indicated on the *y*-axis intercepts of the figure: R^2^ = (0.0, 0238) and Q^2^ = (0.0, −0.450). (**C**) A loadings S-plot, with statistically significant features described to have (p (corr)) of ≥0.5 and covariance of (p1) ≥ 0.5. (**D**) A variable importance in projection (VIP) plot for the OPLS-DA model. Significant ions are indicated by a VIP score >1.0. The computed OPLS-DA was significant, validated by a *p-*value = 7.92 × 10^−7^.

Supervised OPLS-DA models were constructed that use prior class information. In this case, treated samples and control samples were designated as two different groups (Figure 3A). The sample groups (Control_12 h vs. MeSA_12 h) formed a clear separation in the scores space and this indicates the difference in the molecular composition of the samples (control vs. treated) and that these differences are responsible for the group separations observed in Figure 3A [37]. The significance of the OPLS-DA model was estimated using a response permutations test (Figure 3B), where 50 random permutations were performed and R^2^ and Q^2^ values were obtained. These values (R^2^ and Q^2^) were compared to those of the generated OPLS-DA models. The permutated models resulted in the R^2^ = (0.0, 0238) and Q^2^ = (0.0, −0.450), which is lower than that of the originally computed OPLS-DA model (R^2^ = 0.598 and Q^2^ = 0.989) indicating that the original is a statistically viable model. CV-ANOVA was also used to determine the significance of the model. A *p-*value of <0.05 denotes a good model [37,38], and the *p-*value of the computed OPLS-DA model was significant at 7.92 × 10^−7^. Following supervised multivariate statistical analysis, significant ions (with VIP scores ≥1) were putatively annotated based on accurate mass, Rt and MS fragmentation patterns and compared against literature as shown in Table 1 (abbreviations listed in the table). Similar supervised statistical analysis was performed comparing control_12 h vs. MeJA_12 h (Figure S1), control_24 h vs. MeSA_24 h and (Figure S2), control_24 h vs. MeJA_24 h (Figure S3) as shown in the supplementary material.

### 2.3. Comparative Analysis of Metabolites Identified in Leaves Treated with MeSA and MeJA

The rich phytochemical composition of *B. pilosa* is evident from the annotated metabolites (Table 1) that include HCA derivatives of quinic acid and tartaric acid as well as flavonoids [8,39,40]. These include HCA conjugates to tartaric acid (caftaric and chicoric acids) and quinic acid (mono- and di-caffeoylquinic acids), as well as glycosylated and acetylated derivatives of flavonoids such as quercetin, kaempferol, and a tetrahydroxyflavanone.

Individually, MeJA and MeSA are known to regulate a number of physiological processes (i.e., growth and defense responses). As mentioned, MeJA and MeSA have been reported to be important signal molecules in systemic responses of plants to pathogens [41]. Recently they have been used as elicitors to enhance the production of secondary metabolites in medicinal plants and to induce defense responses in crop plant [42]. When applied for this purpose to *B. pilosa*, both MeJA and MeSA treatments were found to exert influence on similar metabolic pathways, as most of the differentially induced metabolites were annotated as CGAs or flavonoids. Elicitors or plant treatment agents have been found to induce similar pathways leading to overlapping responses, with the metabolic pools of certain classes of metabolites more responsive to a specific stimulus [42,43].

**Figure 4:**
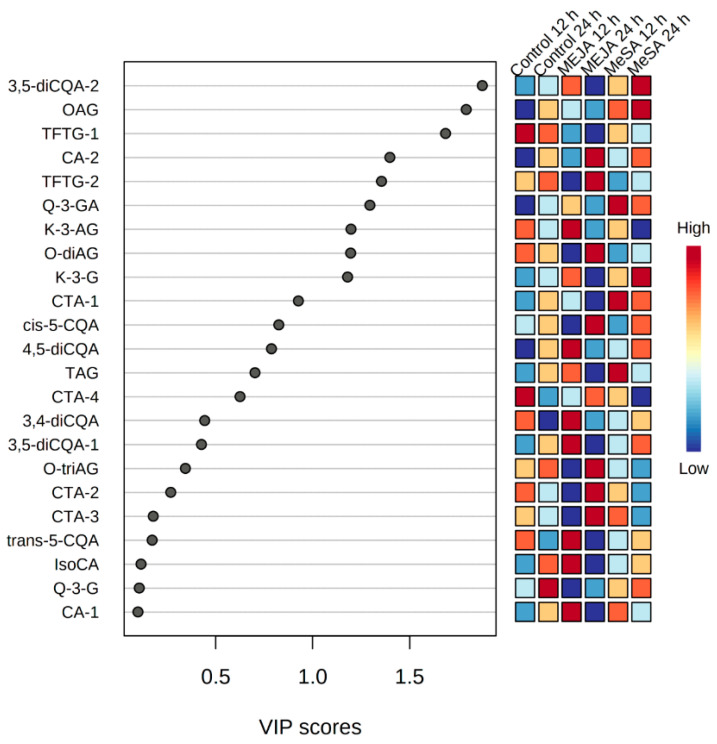
Variable importance in projection (VIP) scores generated from partial least squares discriminant analysis (PLS-DA). Indicated are discriminating ions leaves of *Bidens pilosa* treated with 0.5 mM MeSA and MeJA and harvested at 12 h and 24 h time intervals in comparison to the corresponding control samples. Metabolites with a VIP score ≥0.5 were considered to be affected by exogenous treatments with MeJA and MeSA. Abbreviations are as listed in Table 1.

The stimulating effects of MeSA and MeJA on the *B. pilosa* metabolomes were nuanced in that the OPLS-DA modeling indicated an (i) increase for some of the metabolites, (ii) interconversion between isomeric versions of the same metabolite and (iii) graded differences between the perturbed metabolomes. As apparent from Table 1, both MeSA and MeJA lead to the changes in metabolites of similar pathways in *B. pilosa.* However, as indicated by the profiles of relative changes as shown in Figure 4 (and summarized by the PC and HC analyses), these elicitors induced differential responses to metabolites of the phenylpropanoid pathway.

Earlier studies have demonstrated that both hormones lead to the activation of the phenylpropanoid pathway in various plants and this is associated with enhanced activity of phenylalanine ammonia lyase (PAL), a key enzyme for the synthesis of phenolic compounds. Relatedly, a previous study from our laboratory demonstrated that treatment of tobacco cells with different elicitors (including SA and MeJA) lead to activation of similar pathways but with different metabolic profiles [42].

A time-course analysis of the annotated metabolites in treated leaf extracts that significantly contributed to variability was visualized by means of a VIP scores plot (Figure 4) derived from PLS-DA analysis performed in MetaboAnalyst, which summarizes the weighted sum of squares of the PLS loading, factoring in the explained Y-variance of each component [44]. Here, metabolites with a VIP score of >0.5 were selected as significantly perturbed due to the treatments.

In general, the phenylpropanoid pathway is responsive to both SA and JA signaling, e.g., MeSA was shown to induce activity of PAL, an entry point enzyme to the phenylpropanoid pathway [45], and MeJA was shown to increase the phenolic acid content (including HCAs) by upregulation of the phenylpropanoid pathway in *Brassica oleracea* [46]. Due to cross-talk between the salicylate and jasmonate signaling pathways [47], the effect of JA and SA and derivatives thereof on plant tissues can be unpredictable. For example, SA did not have an effect on the accumulation of phenolics in buckwheat, *Fagopyrum esculentum* [48], and, relatedly, the biosynthesis of CGAs was not induced in *Centella asiatica* cells when treated with SA [49].

Among the differentially perturbed *B. pilosa* metabolites were HCA derivatives, identified as significant ions in response to treatment with either elicitor. In previous studies, these groups of secondary metabolites were reported to have various health benefits such as anti-cancer properties [50], HIV-integrase inhibition by 3,5-di-caffeoylquinic acid [51] and chicoric acid [52] and anti-diabetic properties [53,54]. In this study, the di-caffeoylquinic acids, the 4,5-, 3,4- and 3,5- geometric isomers exhibited similar distribution profiles, prominent in the MeJA_12 and the MeSA_24 conditions. This profile was also broadly applicable to the *trans*-5-CQA that was present in the controls and induced by the MeJA_12 treatment.

Interestingly, *cis*-5-CQA, absent under the control conditions, was indicated as a signatory marker of the MeJA treatment at 24 h, suggesting a role in the stress response. Previously plants were known to only naturally synthesize *trans*-5-CGA, while *cis*-5-CGAs were thought to originate in response to UV-radiation. However, in a recent study, evidence of a possible enzymatic pathway for production of *cis*-CGAs in tobacco regardless of UV radiation was suggested [55].

Other related HCA derivatives included chicoric acid where a time-dependent interconversion was observed. While isomer 1 (CA-1) was annotated as a marker of the control condition, CA-1 was prominent at 12 h and CA-2 at 24 h of treatment with both MeSA and MeJA. In the case of the caftaric acid isomers, CTA-4 was associated with the control condition, while CTA-1, 2, and 3 were found in extracts from leaves treated with MeSA for 12 h with CTA-2 and -3 prominent in extracts of the MeJA_24 treatment.

Flavonoids, which have similarly been identified as significant ions in response to the treatments with the elicitors, were also differentially regulated, as observed for tetrahydroxyflavanone triacetylglucoside isomer 1 (TFTG-1). This flavonoid was relatively higher in control samples as opposed to treatment with MeJA where reduced levels of the metabolite occurred. The same was observed for the MeSA treatment, but to a lesser extent. This corresponds to a report on the treatment of *Calendula officinalis* with MeJA, which resulted in a decrease in flavonoid content [22]. In contrast, the chalcone, okanin di-acetylglucoside (O-diAG) was significantly increased in extracts from leaves treated with MeJA harvested at 24 h and reduced by the equivalent MeSA treatment. MeJA has previously been reported to be positively correlated with the accumulation of flavonoids [56], similar to results from this study for O-diAG at 24 h and for kaempferol-3-O-glucoside (K-3-G) and kaempferol-3-acetyl-glycoside (K-3-AG) at 12 h. Accumulation of flavonoids has been shown to be positively correlated with exogenous treatment of tea (*Camellia sinensis*) leaves with MeSA, where a time-course study showed an increase in flavonoid content from as early as 12 h after treatment. This corresponds to the observation in Figure 4 where accumulation of quercetin-3-O-glucuronide (Q-3-GA) was observed at the 12 h and 24 h time intervals.

Besides flavonoids and HCAs, a stress metabolite/signal molecule, tuberonic acid glucoside (TAG or 12-hydroxyjasmonic acid glucoside), was identified in leaf samples from plants treated with MeSA and harvested at 12 h, and to a lesser extent, MeJA at 12 h. This could indicate a cross-talk of JA- and SA-dependent defense signaling pathways in *B. pilosa* [47], since TAG is important in the response of plants to wounding which is JA responsive [57].

Most metabolites identified in leaf extracts treated with MeSA and MeJA play vital roles in the SAR/ISR responses of plants. Apart from the metabolites mentioned above, treatment with the hormones also affected the primary metabolism as citric acid/isocitric acid, a tri-carboxylic acid (TCA), was also identified as a significant ion, upregulated in MeJA-treated leaves harvested after 12 h. This corresponds to a study performed on *Brassica nigra* where, in response to stress induced by MeJA, regulation of the TCA cycle supported energy requirements for biosynthesis of defense molecules [58]. This indicated upregulation of a primary metabolite showing energy requirements in plants during biosynthesis of defense molecules.

Various studies have reported that treatment of plant tissues/cells with MeJA and MeSA lead to differential regulation of secondary metabolites. These metabolite classes include phenolics such as HCAs and flavonoids [59,60,61]. Treatment of *Lactuca sativa* [60] and *Myrica rubra* (Chinese bayberry) with 0.1 and 1 mM MeJA lead to an increase of total phenolic content due to the enhanced activity of PAL. Similarly, MeJA (0.1 and 0.5 mM) caused a significant increase of the total phenolic content in *Ocimum basilicum* that included rosmarinic acid and caffeic acid [24]. In tea (*C. sinensis*) leaves, MeSA was found to enhance the biosynthesis of flavonoids by stimulating the phenylpropanoid pathway. In that study, time-dependent accumulation of flavonoids was observed to reach a peak 2 d post-treatment with 1 mM MeSA and followed by gradual decrease thereafter, to a concentration lower than the control plants after 6 d [45]. Consistent with the time-course of flavonoid concentration, transcriptome results showed that MeSA activates PAL gene expression, as early as 12 h after the treatment, which peaked after 1 d and then gradually declined over 6 d. Also, MeSA treatment lead to the upregulated expression of genes (such as chalcone synthase, chalcone isomerase, flavanone 3-hydroxylase and dihydroflavonol-4-reductase) involved in flavonoid biosynthesis [45].

## 3. Materials and Methods

### 3.1. Plant Material and Treatment with MeJA and MeSA

*B. pilosa* was grown from seeds in germination mix soil (Culterra, Muldersdrift, South Africa) in a greenhouse at 28 °C with cycles of 16 h light/16 h dark for 8 weeks (~35 cm in height) as previously described [8]. Plants were removed from the greenhouse, and treatments were separately performed due to the volatile nature of the elicitors. Groups of three plants (biological replicates) were treated with 0.5 mM concentrations of MeJA or MeSA (Sigma-Aldrich, München, Germany). An additional group of three replicates were reserved as non-treated controls (NTC). Solutions of MeJA and MeSA were prepared with 8 mM MgSO_4_. Plants for treatment were pressure infiltrated with 1 mL blunt-end syringes with a solution containing the MeJA or MeSA, while the NTC was pressure infiltrated with a solution containing 8 mM MgSO_4_, excluding the signaling molecules. Separate controls were included in the experimental planning to accommodate diurnal changes within the plant that were not associated with the treatments. Leaf material was harvested at 12 h and 24 h post-treatment time-points. The time intervals of 12 h and 24 h were selected to widen the ‘detection window’ to accommodate as many metabolites as possible. NTC plants were similarly harvested at the same time intervals. The leaf materials were immediately placed in liquid nitrogen to quench metabolic activity and stored at −80 °C until metabolite extractions were performed.

### 3.2. Metabolite Extraction

Two grams (2 g) of the frozen leaf tissues were crushed using a mortar and pestle and liquid nitrogen. Pulverized tissue was further homogenized and extracted in 20 mL (1:10 *m*/*v*) of 80% aqueous methanol (Romil SpS, Cambridge, UK). Homogenates were sonicated for 30 min at 30 °C and 100% intensity in a sonicator bath (Branson CPX, Fischer Scientific, Waltham, MA, USA). The crude extracts were centrifuged at 5100 rpm in a benchtop centrifuge (Beckman Coulter, Midrand, South Africa) for 15 min. A rotary evaporator (Heidolph Instruments, Schwabach, Germany) was used to evaporate supernatants, at 55 °C under vacuum to approximately 1 mL samples. These samples were dried overnight in a dry bath at 55 °C. The dried samples were reconstituted with 500 µL of 50% methanol and sonicated for 30 min at 30 °C. Resuspended samples were filtered into HPLC sample vials fitted with 500 µL inserts using 0.22 µm nylon filters. Samples were stored at 4 °C until further analysis. Three independent biological replicates were prepared, and three instrumental technical replicates were analyzed.

### 3.3. Ultra-High Performance Liquid Chromatography-Quadrupole Time-of-Flight Mass Spectrometry (UHPLC-qTOF-MS)

The hydromethanolic extracts were analyzed on an ultrahigh-performance liquid chromatography-quadrupole time-of-flight MS instrument (UHPLC-qTOF SYNAPT G1 system, Waters Corporation, Manchester, UK) with an Acquity HSS T3 C18 column (150 mm × 2.1 mm with particle size of 1.7 μm) (Waters, Milford, MA, USA). A binary solvent gradient [8] was followed to separate extracts. The chromatographic effluents were analyzed utilizing a SYNAPT G1 q-TOF high definition mass spectrometer with electrospray ionization (ESI) (Waters Corporation, Manchester, UK). To obtain fragmentation information of the ionized analytes, fragmentation experiments (MS^E^) were performed by ramping the collision energy from 15 to 60 eV [61]. An in-source collision-induced dissociation (ISCID) method was used to generate data for the annotation of closely related positional isomers of CGAs [8].

### 3.4. Data Processing, Multivariate Data Analysis (MVDA) and Identification of Discriminant Ions

MassLynx XS™ software (Waters, Manchester, UK) was used to extract raw UHPLC-qTOF-MS data which was processed using MarkerLynx XS™ software (Waters, Manchester, UK) prior to MVDA. The following parameters were used: retention time (Rt) range of 0.60–21 min with a Rt window of 0.5 min, mass range of 100–1100 Da and the mass tolerance as 0.05 Da. The data matrix obtained post-processing was exported into SIMCA-15.0 software (Umetrics Corporation, Umea, Sweden) for statistical modeling. Prior to performing principal component (PC) analysis, hierarchical cluster (HC) analysis, and orthogonal projection to latent structures discriminant analysis (OPLS-DA), data was Pareto-scaled. Generated OPLS-DA models were validated by a response permutations test and cross-validated analysis of variance (CV-ANOVA). Potential biomarkers of different sample groups were highlighted in the OPLS-DA S-plot, and only significant biomarkers with [p(corr)] of ≥0.5 and covariance of (p1) ≥0.05 were annotated/putatively identified [38]. To aid in metabolite annotations, fragmentation patterns obtained from MS^E^ experiments (with collision energies from 15 to 60 eV) were used. The accurate mass as generated by the SYNAPT G1 qTOF system was used to derive empirical formulae. These were searched against online databases such as ChemSpider (www.chemspider.com) [62], and Dictionary of Natural Products (www.dnp.chemnetbase.com) [63] and compared to the available literature. Details on the annotation of the metabolites were as previously reported [8]. Metabolites were annotated to level 2 (putatively identified) of the Metabolomics Standards Initiative (MSI) guidelines [64].

### 3.5. Comparative Analysis of Biomarkers

MetaboAnalyst 3.0 (www.metaboanalyst.ca) was utilized to perform partial least square-discriminant analysis (PLS-DA) [65], for visualization and comparison of the relative abundance representing the dynamic changes in the concentrations of the identified metabolites. Peak intensities of discriminatory ions/identified metabolites were imported for exploratory statistical analysis into MetaboAnalyst. Data was normalized and the data was log transformed. This was followed by Pareto-scaling of data sets, performed to reduce systematic variance within features [66]. The partial least square-discriminant analysis (PLS-DA) was computed to mine data of *B. pilosa* leaves treated with MeJA and MeSA. This was performed to investigate the time-dependent responses of treated plants in comparison to NTCs.

## 4. Conclusions

MeJA and MeSA are important endogenous signal molecules, which induce and modulate stress responses in plants. Exogenous application of these signal molecules has been shown to alter the metabolome of plants by increasing production of secondary metabolites, often in a defense-related context. In the current study, MeJA and MeSA were used as elicitors with the aim to evaluate possible enhancement of the biosynthesis of health-beneficial secondary metabolites in *B. pilosa*. Metabolomic profiling of the extracted phytochemicals from the leaves treated with MeJA and MeSA indicated differential responses as reflected by perturbations to the metabolomes. Both treatments were shown to enhance levels of some HCA conjugates to tartaric acid (caftaric- and chicoric acids) and quinic acid (mono- and di-caffeoylquinic acids). Of special interest is the annotation of two isomers of 3,5-di-CQA, two isomers of 5-CQA, four isomers of caftaric acid, and two isomers of chicoric acid. This might be a reflection of *de novo* synthesis, but also the molecular dynamics and perturbed equilibria between the isomers due to the stress response. As seen from the annotated metabolites, most of the isomers were identified as possible *cis* isomers, suggesting a possible role of geometrical isomers as biologically active molecules rather than being mere structural artefacts of their *trans-*isomers as previously thought. In addition, increased levels of the annotated flavonoids, quercetin, and kaempferol were observed in the treated plants.

The observed alterations in the phenolic compound profile in *B. pilosa* leaves can be explained through the actions of MeSA/SA and MeJA/JA, known inducers of gene transcription of PAL and cinnamate 4-hydroxylase, leading into the phenylpropanoid pathway. Since MeJA and MeSA both affected similar metabolites originating from the phenylpropanoid pathway, this might be an indication of an inter-connected response in *B. pilosa* to these phytohormones. Time-dependent differences were also observed in the metabolite levels, signifying the transient nature of the induced responses and suggesting stringent endogenous control systems that return the levels of the metabolites associated with the perturbed metabolomes to homeostatic conditions.

As observed in this study, the metabolome of *B. pilosa* is affected by exogenous treatment with signal molecules used as elicitors. These results add novel insights on the metabolomic responses induced by external stimuli on tissues of *B. pilosa.* This highlights the possibility of using MeJA and MeSA (or inducing agents and stimuli acting through these signal molecules) to elicit the production of important bioactive secondary metabolites, such as chlorogenic acids and flavonoids. Future studies based on these results can allow for optimization of elicitor concentrations, elicitation time, and harvest time, to permit the accumulation of the secondary metabolites.

## Figures and Tables

**Table 1:** List of statistically significant and discriminatory ions (derived from OPLS discriminant analysis) and putative annotation of metabolites present in leaf extracts of *Bidens pilosa* and correlated with the 0.5 mM MeSA or MeJA treatments. All ions (*m/z*) had a VIP score >1.0 and were annotated as described [8], to MSI-level 2.

Rt (min)	Ions *m/z*	Fragment Ions	Molecular Formula	Metabolite	Abbreviation *
1.01	191.019	155, 111	C_6_H_8_O_7_	Citric acid/Isocitric acid	IsoCA
4.64	311.037	179, 146	C_13_H_12_O_9_	Caftaric acid isomer 1	CTA-1
5.57	353.084	191	C_16_H_18_O_9_	*trans*-5-Caffeoylquinic acid	*trans*-5-CQA
7.96	311.074	179, 149	C_13_H_12_O_9_	Caftaric acid isomer 2	CTA-2
8.07	353.085	191	C_16_H_18_O_9_	*cis*-5-Caffeoylquinic acid	*cis*-5- CQA
8.6	311.072	179, 146	C_13_H_12_O_9_	Caftaric acid isomer 3	CTA-3
8.85	387.163	207, 163	C_18_H_28_O_9_	Tuberonic acid glucoside	TAG
9.31	311.072	179,149	C_13_H_12_O_9_	Caftaric acid isomer 4	CTA-4
12.69	491.116	287, 151	C_23_H_24_0_12_	Okanin acetylglucoside	OAG
13.51	477.065	301	C_21_H_18_O_13_	Quercetin-3-O-glucuronide	Q-3-GA
13.71	463.086	301	C_21_H_19_O_12_	Quercetin-3-glycoside	Q-3-G
14.10	515.120	353, 335, 191, 173, 135	C_25_H_24_O_12_	3,4-di-Caffeoylquinic acid	3,4-di-CQA
14.32	515.118	353, 191, 179, 135	C_25_H_24_O_12_	3,5-di-Caffeoylquinic acid isomer 1	3,5-di-CQA-1
14.49	447.089	285	C_27_H_30_O_15_	Kaempferol-3-O-glucoside	K-3-G
14.62	515.116	353, 191, 179, 135	C_25_H_24_O_12_	3,5-di-Caffeoylquinic acid isomer 2	3,5-di-CQA-2
14.95	473.073	311, 179, 149	C_22_H_18_O_12_	Chicoric acid isomer 1	CA-1
15.22	515.117	353, 91, 179, 173, 135	C_25_H_24_O_12_	4,5-di-Caffeoylquinic acid	4,5-di-CQA
15.43	473.071	311, 179, 149	C_22_H_18_O_12_	Chicoric acid isomer 2	CA-2
15.99	489.086	285	C_23_H_22_O_12_	Kaempferol-3-acetyl-glycoside	K-3-AG
17.60	533.128	287, 151, 135	C_25_H_26_O_13_	Okanin di-acetylglucoside	O-diAG
17.94	575.139	287, 135	C_27_H_28_O_14_	Okanin tri-acetylglucoside	O-triAG
19.16	575.138	285, 135	C_27_H_28_O_14_	Tetrahydroxy-flavanone triacetylglucoside isomer 1	TFTG-1
19.52	575.138	285, 135	C_27_H_28_O_14_	Tetrahydroxy-flavanone triacetylglucoside isomer 2	TFTG-2

* Metabolite abbreviations relate to those of Figure 4 where relative intensities of metabolites are presented in a heat map format.

## Supplementary Materials

The following are available online at https://www.mdpi.com/2223-7747/9/10/1275/s1, Figure S1: An orthogonal projection to latent structures discriminant analysis (OPLS-DA) model computed of control and 0.5 mM MeJA treated leaf extracts at the 12 h time point post-treatment. Figure S2: An orthogonal projection to latent structures discriminant analysis (OPLS-DA) model computed of control and 0.5 mM MeSA treated leaf extracts at the 24 h time point post-treatment. Figure S3: An orthogonal projection to latent structures discriminant analysis (OPLS-DA) model computed of control and 0.5 mM MeJA treated leaf extracts at the 24 h time point post-treatment.
