# Supplementary material for: Profiling of Altered Metabolomic States in Bidens pilosa Leaves in Response to Treatment by Methyl Jasmonate and Methyl Salicylate
Source: Plants (Basel). 2020 Sep 27;9(10):1275. doi: 10.3390/plants9101275 (PMC7601133; doi:10.3390/plants9101275)
Supplement: Supplementary file 1 [file plants-09-01275-s001.pdf]

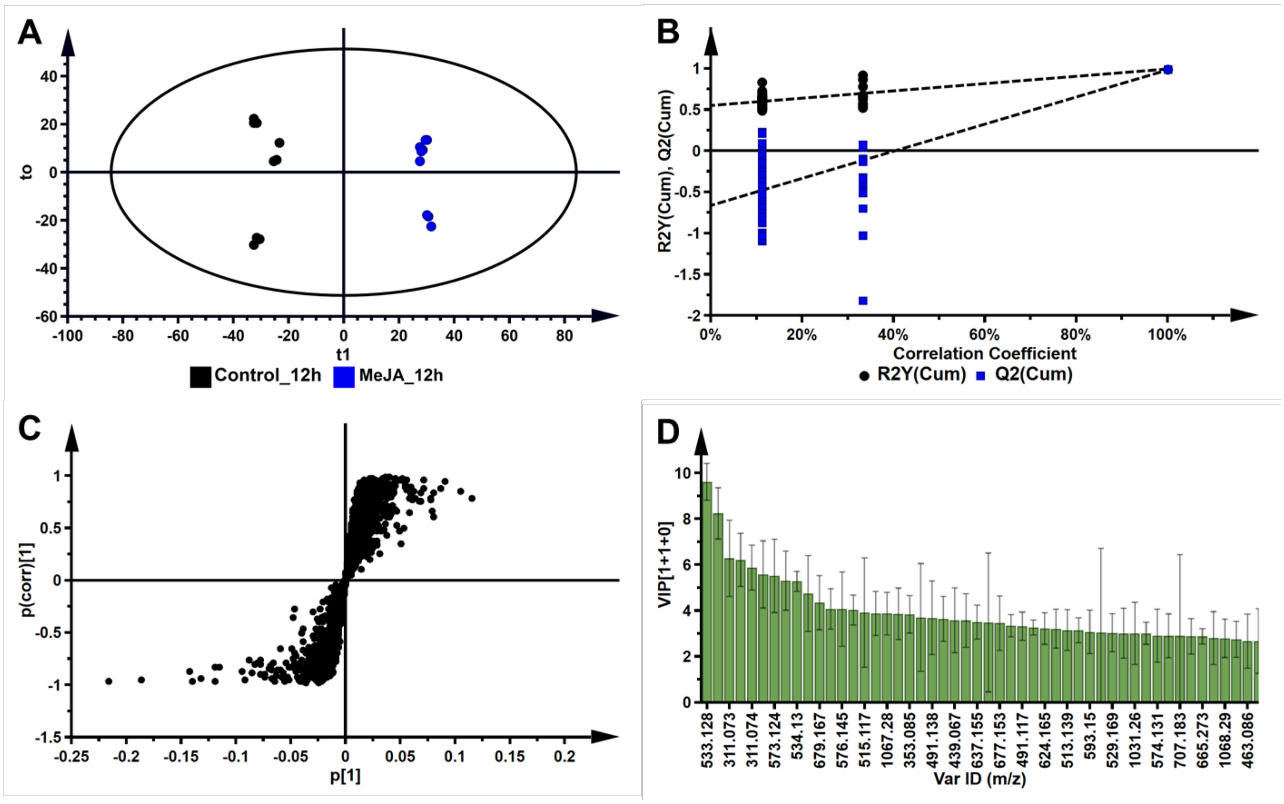

Figure S1. An orthogonal projection to latent structures discriminant analysis (OPLS-DA) model computed of control (orange) and 0.5 mM MeJA (grey) treated leaf extracts at the 12 h time point post treatment. (**A**) A score plot summarising the relationship between the two conditions. (**B**) A permutation test plot (*n*=50) in response to the OPLS-DA plot indicated in **A**, with the quality parameters indicated on the y-axis intercepts of the figure: R2= (0.0, 0.891) and Q2= (0.0, -0.502). (**C**) A corresponding loadings S-plot, with statistically significant features described to have [P(corr)] of ≥ 0.5 and covariance of (p1) ≥ 0.5. (**D**) A variable importance in projection (VIP) plot for the OPLS-DA model of samples from plants treated with 0.5 mM MeJA. Ions that are responsible for the significant separation observed between the two sample groups are indicated by a VIP score greater than 1.0. The computed OPLS-DA was significant, validated by a *p*-value= 7.03×10^{-11} .

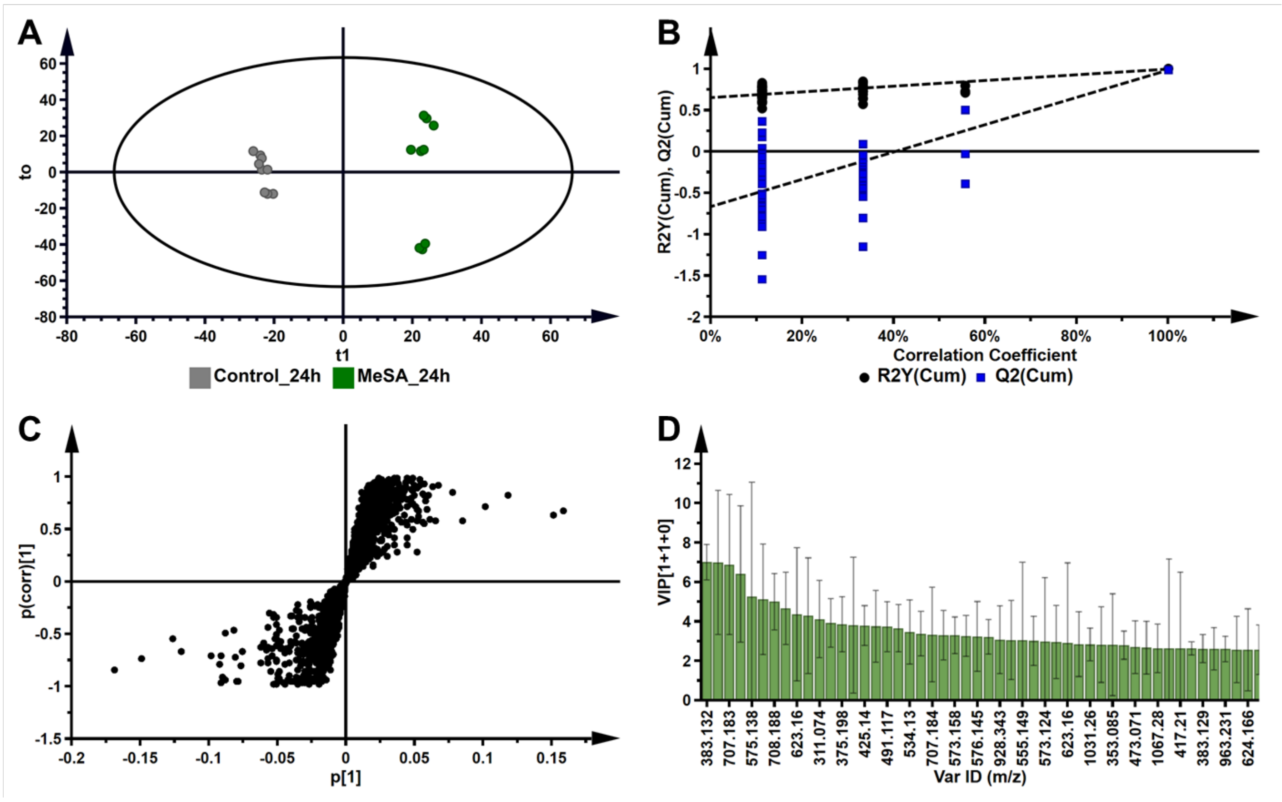

Figure S2. An orthogonal projection to latent structures discriminant analysis (OPLS-DA) model computed of control (pink) and 0.5 mM MeSA (light blue) treated leaf extracts at the 24 h time point post treatment. (**A**) A score plot summarising the relationship between the two conditions. (**B**) A permutation test plot (*n*=50) in response to the OPLS-DA plot indicated in **A**, with the quality parameters indicated on the y-axis intercepts of the figure: R2= (0.0, 0.651) and Q2= (0.0, -0.668). (**C**) A corresponding loadings S-plot, with statistically significant features described to have [P(corr)] of ≥ 0.5 and covariance of (p1) ≥ 0.5. (**D**) A variable importance in projection (VIP) plot for the OPLS-DA model of samples from plants treated with 0.5 mM MeSA. Ions that are responsible for the significant separation observed between the two sample groups are indicated by a VIP score greater than 1.0. The computed OPLS-DA was significant, validated by a *p*-value= 3.21×10^{-11} .

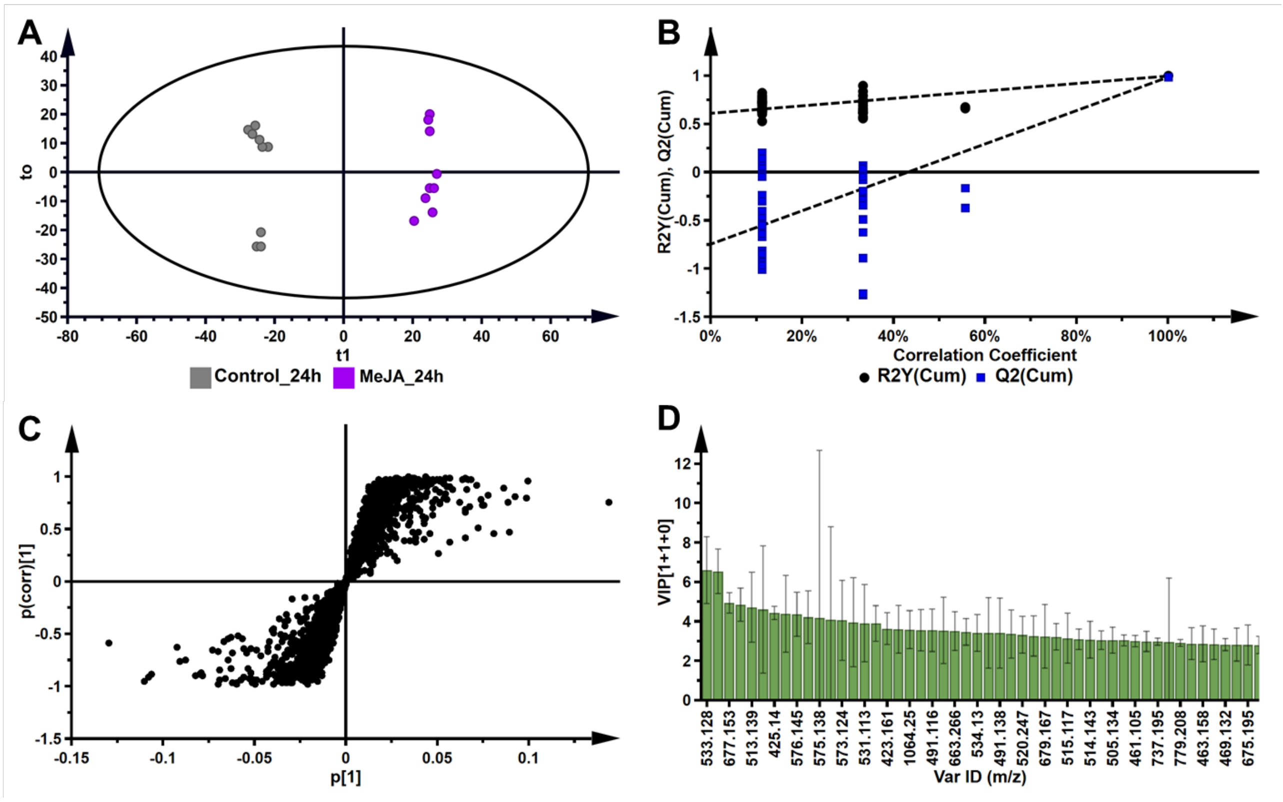

Figure S3. An orthogonal projection to latent structures discriminant analysis (OPLS-DA) model computed of control (pink) and 0.5 mM MeJA (yellow) treated leaf extracts at the 24 h time point post treatment. (**A**) A score plot summarising the relationship between the two conditions. (**B**) A permutation test plot (*n*=50) in response to the OPLS-DA plot indicated in **A**, with the quality parameters indicated on the y-axis intercepts of the figure: R^2= (0.0, 0.609) and Q^2= (0.0, -0.747). (**C**) A corresponding loadings S-plot, with statistically significant features described to have [P(corr)] of ≥ 0.5 and covariance of (p1) ≥ 0.5. (**D**) A variable importance in projection (VIP) plot for the OPLS-DA model of samples from plants treated with 0.5mM MeJA. Ions that are responsible for the significant separation observed between the two sample groups are indicated by a VIP score greater than 1.0. The computed OPLS-DA was significant, validated by a *p*-value= 2.63×10^{-11} .
